# Transient Cold Hemagglutinin Disease (CHAD) During Cardiac Surgery: An Unexpected Complication from Hypothermia

**DOI:** 10.34172/jcvtr.33536

**Published:** 2026-06-30

**Authors:** Andrea D’Alessio, Ali Ansaripour, Samail Shahjahan, Joshua Oliver, Charles Chan, Vivek Srivastava

**Affiliations:** Departement of Cardiothoracic Surgery, Oxford University Hospitals NHS Foundation Trust, Oxford, UK

**Keywords:** Cold hemagglutinin disease, Cardiac surgery, Intraoperative complications, Autoimmune haemolytic anaemia, Hypothermia

## Abstract

A 74-year-old male with mild non-specific anaemia underwent elective mitral and tricuspid valve surgery. Following initiation of cardiopulmonary bypass (CPB), systemic cooling to 34℃ and cardioplegia at 4℃, upon cardiotomy, clumps of blood were noted in the left atrium and left ventricle. ACT was confirmed to be in range, therefore a blood sample from CPB machine was collected and when exposed to ice demonstrated clumps, while they disappeared once at normal temperature. Hence, the patient was rewarmed to 37℃, subsequent cardioplegia doses were delivered warm and the heart chambers were washed out. Mitral and tricuspid valve repair were carried out uneventfully and the patient made an uncomplicated recovery. Positive direct antiglobulin test confirmed CHAD, a form of autoimmune haemolytic anaemia involving IgM autoantibodies against red blood cells triggered by low temperature. The prompt intraoperative identification and subsequent modifications of surgical strategy potentially averted severe embolic and ischemic complications.

## Introduction

 Cold hemagglutinin disease is a rare type of autoimmune haemolytic anaemia distinguished by IgM autoantibodies against red blood cells (RBC). These autoantibodies are triggered by low temperatures, leading to destruction of RBC. The mechanism is initiated by cold agglutinins (CA) that bind to antigens located on the outer membrane of erythrocytes, and activation of the complement system. This phenomenon leads to agglutination, a process that ultimately culminates in haemolysis and development of blood clots. Cardiac surgery frequently employs hypothermia and poses a risk for individuals with CHAD and therefore requires caution.^1^

## Case Presentation

 A 74-year-old male was planned for mitral and tricuspid valve repair for severe primary mitral regurgitation and secondary tricuspid regurgitation with preserved biventricular function. Co-morbidities included remote myocardial infarction with stenting, controlled hypertension and asthma. During preoperative assessments, coronary angiogram confirmed stents patency, mild normochromic normocytic anaemia with normal reticulocyte was identified (haemoglobin 113 g/L, MCV 95.2 fL, MCHC 336 g/dL, reticulocyte 2,1%, normal iron and transferrin), liver and kidney function were also normal (ALT 25 IU/L, total bilirubin 22 umol/L, Creatinine 79 umol/L) with unremarkable clotting. No blood group antibodies were identified prior to surgery. Hence, no further tests were indicated, given no concerns for bleeding or coagulation abnormalities.

 At surgery, sternotomy was performed, 400-500 Unit/Kg of heparin was administrated and ACT > 480 sec was achieved. CPB via ascending aorta and bicaval cannulation, systemic cooling to 34º C was commenced. Antegrade cold blood cardioplegia at 4º C was delivered to achieve cardiac arrest. The left atrium was opened and as the mitral valve was being analysed, blood clumps were noted in the left atrium and ventricle. ACT was confirmed to be in range. A sample of blood from CPB machine was collected in a syringe and exposed to ice to lower its temperature. This demonstrated blood clumps formation in the syringe and on rewarming of this sample, the clumps were seen to disappear (Figure 1), raising suspicion of cold agglutinins. Haematological consultation was sought and no additional rapid intraoperatively diagnostic tests were available.

**Figure 1:**
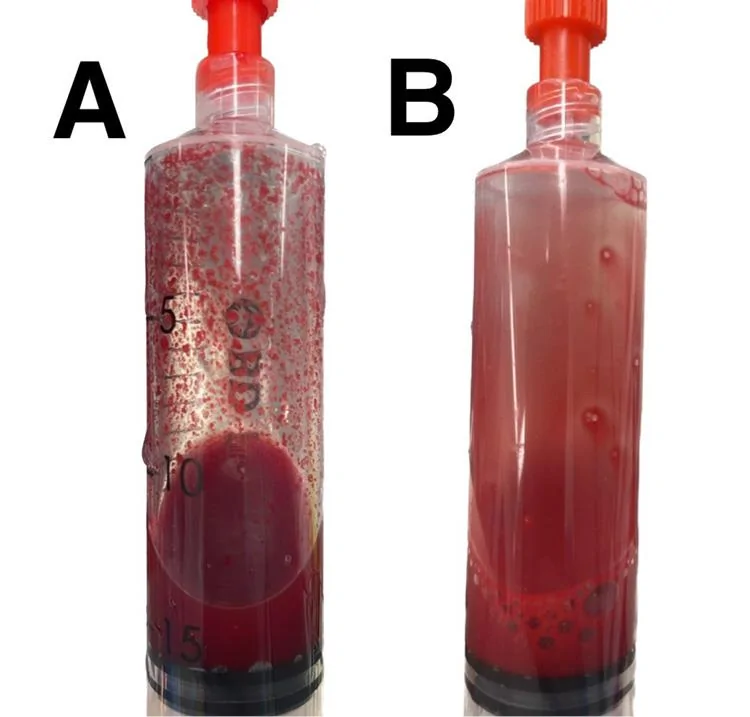
A) Syringe of blood sample from the patient showing clumping following ice exposure; B) same sample at rewarming demonstrate dissolution of clumps

 To reduce the likelihood of agglutination and associated risks, the patient was immediately rewarmed to normothermia, the following doses of cardioplegia were delivered warm and warm saline was used to wash out the heart chambers. After mitral and tricuspid valve were repaired, warm reperfusion was initiated and the operation was completed without major complications. The patient remained haemodynamically stable and he was extubated on the same day. He initially developed blood-stained urine, received RBC transfusion for anaemia and bilirubin peaked at 32 umol/L. Haematological screening tests were performed and a positive direct antiglobulin test (Coombs test) confirmed the diagnosis. Otherwise, the patient made an uncomplicated recovery and was discharged in excellent health.

## Discussion

 CHAD comprises a range of haemolytic anaemias that share comparable clinical presentations despite their distinct aetiologies. CHAD can be classified as either cold agglutinin disease (CAD), a lymphoproliferative disorder affecting B-cells from the blood and bone marrow, or cold agglutinin syndrome (CAS), which is secondary to infections (like COVID-19 and other), cancers or autoimmune diseases.^1^

 Erythrocytes present I/i and Pr antigens on the surface besides ABO. IgM antibodies (CA) are expressed towards those antigens and activated by hypothermia, altering RBCs, which become more adherent to the periphery and clumps agglutinate. This process is reversible with rewarming, as RBC dissociates from CA and the aggregate dissolves. Agglutinated RBCs undergo C3b complement fixation, which remain bonded also after rewarming. This complex gets destroyed in the liver leading to extravascular hemolysis or activate complement mediated intravascular hemolysis. During rewarming, C3b on surviving RBCs is degraded into inactive C3d, protecting them from hemolysis and returning to circulation.^1,2^

 Notably, there can be significant variation in autoantibody titers in response to temperature changes. Therefore, a considerable number of cases of CHAD remain undetected until they undergo surgery, and again there may be no symptoms or only transient haemolytic anaemia.^2^ Hence, as in our case, the diagnosis is often made intraoperatively during hypothermic cardiopulmonary bypass, injection of cold blood-based cardioplegia or deep systemic hypothermia. Potentially, blood clots so formed, if not detected and treated quickly, can lead to severe consequences such as myocardial ischemia, embolic events, and widespread haemolytic anaemia.^3^ Therefore, rewarming remains the only treatment available, despite the risk of haemolysis. The complications correlate with antibody titer, thermal amplitude and duration of hypothermia. In our case, a prompt identification of CHAD and swift rewarming mitigated the complications with minor postoperative signs of haemolysis such as anaemia and haematuria.

 Although CHAD can lead to potentially serious complications, the need for routinely testing is a matter for discussion. In the general population, its prevalence is 10 - 16 per million people.^4^ Jain et al. investigated 14,900 patients undergoing cardiac surgery and found a mere 0.3% prevalence of CHAD. Patients with CHAD were treated with adjustments to surgical strategy, and although the duration of hospitalisation and intensive care for this cluster was slightly prolonged, the study did not identify a substantial rise in mortality or morbidity. They concluded that the cost-effectiveness of universal preoperative screening for CHAD remains unclear.^5^

 If, however, patients are suspected or known to have CHAD, Berentsen (2016) recommends comprehensive haematological evaluation prior to surgery. These include blood tests to identify haemolytic anaemia (Hb, MCV, LDH, reticulocyte count, haptoglobin, and bilirubin), direct Coombs test, assessment of autoantibody titer, and thermal amplitude.^2^ However, they are not practical as confirmatory tests if there was suspicion of CHAD intraoperatively.^6^

 There is still considerable uncertainty regarding the management of CHAD in the context of cardiac surgery and proposed strategies differ based on the phase of the patient’s surgical journey. When detected prior to surgery, treatment focuses primarily on mitigating measures to decrease autoantibody titers and determine thermal amplitude. In these cases, Tjønnfjord et al. propose preoperative administration of eculizumab,^7^ whereas alternative research proposes Rituximab or plasmapheresis.^2^ While, when detected intraoperatively, the focus is on reducing agglutination and clot formation to improve patient outcomes.^4^

 Identifications of blood clots during CPB is a rare event and it could be secondary to acquired or genetic heparin resistance, disseminated intravascular coagulation, genetic coagulation defect as Factor V Leiden deficiency or Factor II G20210A mutation, polycythaemia vera and CHAD.

 In our experience, blood clots were incidentally observed during mitral valve assessment while the patient was receiving active systemic cooling and cold cardioplegia. ACT was within range and there was no history or clinical evidence of blood disorder. We employed a rapid testing method with a syringe to establish a provisional clinical diagnosis. To reduce autoantibody activation and dissolve clumps, we reverted to normothermia and switched to warm cardioplegia, which protected against embolic events and minimised haemolytic complications. The reversibility of autoantibodies at normal temperatures highlights the critical role of rewarming procedures.^8^ During normothermia, frequent warm cardioplegia and good systemic perfusion are essential to reduce the risk of ischemia due to increased body metabolism and oxygen consumption.

## Conclusion

 CHAD is an uncommon condition but one with particular relevance to cardiac surgery. Although universal preoperative screening may not be achievable, it is important to maintain a vigilant attitude during surgery for signs of agglutination. Swift intraoperative diagnosis with improvised thermal tests and modification of surgical strategy can avert potentially disastrous consequences.

## Competing Interests

 The authors have nothing to declare

## Ethical Approval

 Informed consent from the patient was obtained for the publication of the case report and accompanying images.
